# DNA variation in the phenotypically-diverse brown alga *Saccharina japonica*

**DOI:** 10.1186/1471-2229-12-108

**Published:** 2012-07-11

**Authors:** Evgeniy S Balakirev, Tatiana N Krupnova, Francisco J Ayala

**Affiliations:** 1 Department of Ecology and Evolutionary Biology, University of California, 321 Steinhaus Hall, Irvine, CA, 92697-2525, USA; 2 A. V. Zhirmunsky Institute of Marine Biology, Far Eastern Branch of the Russian Academy of Science, Vladivostok, 690059, Russia; 3 Pacific Research Fisheries Centre (TINRO-Centre), Vladivostok, 690600, Russia

**Keywords:** Brown algae, Kelp, *Saccharina*, DNA polymorphism, Interspecific *COI* recombination, Phenotypic plasticity, *Saccharina japonica* morphological forms

## Abstract

**Background:**

*Saccharina japonica* (Areschoug) Lane, Mayes, Druehl et Saunders is an economically important and highly morphologically variable brown alga inhabiting the northwest Pacific marine waters. On the basis of nuclear (*ITS*), plastid (*rbc*LS) and mitochondrial (*COI*) DNA sequence data, we have analyzed the genetic composition of typical *Saccharina japonica* (TYP) and its two common morphological varieties, known as the “longipes” (LON) and “shallow-water” (SHA) forms seeking to clarify their taxonomical status and to evaluate the possibility of cryptic species within *S. japonica*.

**Results:**

The data show that the TYP and LON forms are very similar genetically in spite of drastic differences in morphology, life history traits, and ecological preferences. Both, however, are genetically quite different from the SHA form. The two *Saccharina* lineages are distinguished by 109 fixed single nucleotide differences as well as by seven fixed length polymorphisms (based on a 4,286 bp concatenated dataset that includes three gene regions). The GenBank database reveals a close affinity of the TYP and LON forms to *S. japonica* and the SHA form to *S. cichorioides*. The three gene markers used in the present work have different sensitivity for the algal species identification. *COI* gene was the most discriminant gene marker. However, we have detected instances of interspecific *COI* recombination reflecting putative historical hybridization events between distantly related algal lineages. The recombinant sequences show highly contrasted level of divergence in the 5’- and 3’- regions of the gene, leading to significantly different tree topologies depending on the gene segment (5’- or 3’-) used for tree reconstruction. Consequently, the 5’-*COI* “barcoding” region (~ 650 bp) can be misleading for identification purposes, at least in the case of algal species that might have experienced historical hybridization events.

**Conclusion:**

Taking into account the potential roles of phenotypic plasticity in evolution, we conclude that the TYP and LON forms represent examples of algae phenotypic diversification that enables successful adaptation to contrasting shallow- and deep-water marine environments, while the SHA form is very similar to *S. cichorioides* and should be considered a different species. Practical applications for algal management and conservation are briefly considered.

## Background

Genetically-based approaches (especially using multi-locus data) have resolved much phylogenetic uncertainty in large-scale algal trees of life (e.g., [1-3]). Less obvious progress, however, has been accomplished concerning the species level of algal taxonomy, when there is an abundance of morphological variants with uncertain taxonomic status. For instance, the genus *Laminaria* (that has been recently separated into two different genera, *Laminaria* and *Saccharina*; [1]) is rich with morphological types, varieties, ecotypes, and forms that have puzzled taxonomists since the very beginning of research on this subject [4-14]. Whether the distinct morphologies observed in many algae represent different species or only intraspecific forms is one of the main unresolved questions.

In this paper we focus on the most common and commercially important laminarialean species, *Saccharina japonica*[4,5,15] and its morphological forms, inhabiting contrasting depths of the northwest Pacific region. The species is morphologically and ecologically highly variable and some intraspecific forms have been described [10-13]. Typical *S. japonica* (TYP) inhabits the littoral zone at the preferred depths of 5 – 11 m with wide distribution over the full species area. Its full distribution pattern, however, remains uncertain owing to the difficulty of reliable species identification. It grows along the sea shore on rocky substrate [5,6,15-17]. (See Additional file 1 for a more detailed species description.) At present the populations of the TYP form are depressed due to overharvesting and possibly global climate changes [18,19].

The deep-water (or “longipes”) *S. japonica* form (LON) inhabits the sublittoral zone at preferred depths of 14 – 25 m. This endemic form has a restricted distribution in the Sea of Japan and the Sea of Okhotsk. It grows in compact settlements at a significant distance (300 – 1000 m) from the seashore [6,16,17,20,21]. The LON form thallus is 2 – 3 times longer and 3 – 4 times heavier than the thallus of the TYP form. Differences in morphology and preferred ecology are detectable already among algae during the first year of growth. Originally the LON form was described as a deep-water variant of *S. japonica*[4,6]; a new nomenclatural designation, *S. japonica* f. *longipes*, has been suggested recently [13]. The TYP and LON forms have significant differences in morphology, anatomy, reproductive biology, ecology, and other important features (Additional file 1). The opportunity for interbreeding between the LON and TYP forms is considered highly unlikely because the formation, release, and appearance of the mature gametes are separated in space and time. The zoospore spreading time of *S. japonica* (and the close species, *S. angustata*) is short (maximum 24 hours) leading to limited free dispersal gamete range (on average no more than 3.5 meters [19,22]). Also the distribution areas of the LON and TYP forms are separated geographically (not less than three miles from each other) and their reproductive periods do not overlap (Additional file 1). The morphological differences between the TYP and LON forms of *S. japonica* are stable and easily recognizable and they are systematically reported from the northern Primorye coast region [20,21]. The drastic differences between the TYP and LON forms have motivated changing the taxonomical status of the LON form, so that it would be considered a separate species [17]. However, transplant experiments [23,24] show that zoospores of the LON form released in the habitat of TYP form produced algae morphologically indistinguishable from the TYP form, which did not support the biological species status of the LON form, but rather suggested high phenotypic plasticity for the species.

The shallow-water form of *Saccharina japonica* (SHA) inhabits the supralittoral zone (0.1 – 0.5 m depth) and it is widely distributed in the Primorye coast region, the Sea of Japan, including Peter the Great Bay. It is commonly accepted that the SHA form represents an intraspecific ecotype of the TYP *S. japonica* form. The zoospores of the SHA form are used to enhance the strongly overharvested resources of the TYP form [19]. Nevertheless, the SHA and TYP forms have clearly distinct morphological features (Additional file 1). There are also significant differences between the forms in heat and salinity tolerance [25]. The SHA form is more heat and desalination resistant and survives better than the TYP form under hyposalinity osmotic stress and increasing temperature conditions. It was suggested [26] that the SHA form may represent a subspecies of *Laminaria* (*Saccharina*) *angustata* adapted to the supralittoral zone.

We analyze the genetic composition of *S. japonica* and its common morphological forms, LON and SHA, seeking to clarify the taxonomic status of the forms and to evaluate the possibility of cryptic species within *S. japonica*, using DNA nucleotide sequences of three genes from mitochondrial, plastid, and nuclear genomes (4,286 bp total in a concatenated dataset). We also test for homogeneous phylogenetic signal within and among the marker loci (*ITS*, *rbc*LS, and *COI*). We have found that the TYP and LON morphological forms are genetically very similar; consequently they need not be considered as different biological species. However, taking into account the potential evolutionary roles of phenotypic plasticity, and its relevance to adaptive evolution and speciation in many organisms (e.g., [27-29]) including algae (e.g., [30,31]) we propose that the TYP and LON forms may represent an important example of algal diversification that enables successful adaptation to contrasting shallow- and deep-water marine environments. The SHA form has clear genetic differences from the TYP and LON forms and it might represent a morphological variant of *S. cichorioides* expanded to the supralittoral environment. The practical implications for mariculture are that all three forms should be considered separate evolutionary lineages and that restoration programs should include genetic data to avoid confounding.

## Methods

### Algae samples

The specimens of *S. japonica* morphological forms were collected from the Primorye coastal region, Sea of Japan. The TYP (five specimens) and SHA (four specimens) forms were collected near the Cape Dal'niy at depths of 6.0 m and 0.5 m, respectively**.** The LON form (three specimens) was collected near the Cape Zolotoi at depths of 15.0 m. We also analyzed first year individuals belonging to the *S. japonica* typical form (TYPF, four specimens), collected near the Cape Dal'niy; unidentified *Saccharina* species (CHE, four specimens; depth of 0.5 m), TYP form growing in close proximity with unidentified *Saccharina* species (TYPA, four specimens), and a sample of the TYP form without middle line (TYPW, a single specimen), all collected in the Bay Chernoruch'e near the Cape Khitrovo. The algae were identified by eye in the field.

### DNA amplification and sequences

Total genomic DNA was extracted using the DNeasy Plant MiniKit protocol (Qiagen, Hilden, Germany). The procedures for DNA amplification and sequencing have been described previously [32-34]. Plastid sequences for the partial *rbc*LS operon (RubisCO; 1,822 bp total) included 1,333 bp of the flanking *rbc*L gene, the complete spacer (282 bp), and 207 bp of the flanking *rbc*S gene. For the *rbc*LS operon, primers and PCR conditions followed [1]. Mitochondrial sequences of a 1,788-bp *COI* fragment were amplified using newly developed primers (annealing temperature 52°C): 5’-cttatcaaaaggtgcatctatgg-3’ (SacCOIF; forward) and 5’-acactctaccgctgagttacaag-3’ (SacCOIR; reverse) designed based on full mitochondrial genomes of 11 brown algal species [11,35-37]. Our sequences include the full coding region (1,602 bp, 534 codons) of the *COI* gene as well as 108 bp and 78 bp of the 5'- and 3'-flanking regions, respectively. The nuclear 676-bp fragment of the 18 S–ITS1–5.8 S–ITS2 rDNA region (*ITS*) was amplified using primers (annealing temperature 53°C): 5’-aggtccgaacgaaagtggta-3’ (SacITSF; forward) and 5’-acaaggtttccgtaggtgaac-3’ (SacITSR; reverse). The amplified fragment includes partial 18 S ribosomal RNA gene (17 bp), complete ITS1 (244 bp), complete 5.8 S ribosomal RNA gene (159 bp), and partial ITS2 (256 bp). Using all three gene regions we constructed a 4,286-bp concatenated alignment (with alignment gaps) of 25 algae samples. At least two independent PCR amplifications were sequenced for each polymorphic site in all 25 algae samples to correct for possible PCR or sequencing errors. (See Additional file 2 for the PCR details). The *COI*, *ITS*, and *rbc*LS sequences have been deposited in GenBank under accession numbers JN873222-JN873246, JN873247-JN873271, and JN873272-JN873296, respectively.

### DNA sequence analysis

The sequences were assembled using the program SeqMan (Lasergene, DNASTAR, Inc.). Multiple alignment was carried out manually and using the program CLUSTAL W [38]. The computer programs DnaSP, version 5 [39] and PROSEQ, version 2.9 [40] were used for most intraspecific analyses. *Saccharina coriacea*, *S.angustata*, *Laminaria digitata*, and *Agarum clathratum* (see Additional file 3) were used as outgroup taxa; they were selected based on previous molecular evidence of close relationship to *S. japonica*[1,2,11,41-43] and screening of nucleotide sequences available in GenBank.

Model-based phylogeny reconstructions were performed with the *rbc*LS, *COI*, *ITS*, and concatenate sequence alignments using the neighbor-joining (NJ) and maximum-likelihood (ML) methods in MEGA, version 5 [44]. MEGA5 or jModelTest [45] were used to find the best model of substitution under the maximum likelihood criterion. Models with the lowest Bayesian information criterion [46] scores were considered most appropriate to describe evolution for each gene; the Akaike Information Criterion [47] was also applied. Maximum likelihood bootstrap analyses [48] consisted of 1000 replicates.

Partitioned analyses were performed with GARLI, version 2.0 [49] and MrBayes, version 3.2 [50] that allowed the overall rate to be different across partitions (*rbc*LS, *COI*, and *ITS*). Substitution model parameters were estimated separately for each gene. The most appropriate models were Tamura 3-parameter plus gamma for *rbc*LS, Hasegawa-Kishino-Yano plus I for *COI*, and Tamura 3-parameter for *ITS*.

The complete dataset was also analyzed by Bayesian inference using MrBayes, version 3.2 [50] under a general-time-reversible model plus gamma plus I. Proportion of invariable sites was uniformly distributed on the interval (0.00, 1.00). Gamma distribution was approximated using 4 categories. Analyses were performed as two independent runs, each with four incrementally heated Metropolis-coupled Monte-Carlo Markov Chains running for one million generations. Output trees and data were sampled every 500 generations. Likelihood values reached a plateau within approximately 1,000 generations. A total of 4002 trees in two files were read and 3002 of them were sampled. The numbers of unique site patterns were 100, 175, and 107 for *rbc*LS, *COI*, and *ITS*, respectively. It showed that *COI* as the most efficient gene marker consisting with the distance-based methods.

The average standard deviation of split frequencies at the end of the run was 0.0068 indicating stationary conditions. The log likelihood values increased from below −35292.434 to around −9652.102 in the first five thousand generations and then to around −9547.021 after one million generations. The likelihood of best state for “cold” chain of run 1 was −9523.80 and the likelihood of best state for “cold” chain of run 2 was −9524.14. At the end of run there were not any trends in a plot of generation versus the log probability of the observed data (the log likelihood values). A convergence diagnostic, the Potential Scale Reduction Factor (PSRF) [51] was between 1.00 and 1.003 indicating a good sample from the posterior probability distribution.

The topologies obtained with neighbor-joining and maximum-likelihood methods as well as with Bayesian inference were very similar. The close congruence could be explained by the fact that 1) the dataset was relatively straightforward and included just two main compared groups of sequences (TYP and SHA) and four outgroup species (*S. coriacea*, *S. angustata*, *Laminaria digitata*, and *Agarum clathratum*); 2) the extent of sequence divergence between the TYP and SHA forms for all gene regions was less than 5.0% (0.9% for *rbc*LS, 1.1% for *ITS*, and 4.8% for *COI*); and 3) the length of alignment was long enough (4.2 kb totally). As has been shown by many authors, the relative efficiencies of the ML and NJ methods in obtaining the correct tree topology were very close under these conditions [52-54]. The only difference between the two methods in relation to our dataset concerned slightly different bootstrap values. To be conservative we show the lowest bootstrap values obtained with the ML method. When different methods produced similar or identical topologies the simplest, NJ method, was preferable.

## Results

### Nucleotide diversity and divergence

We sequenced three gene regions (total 4,286-bp alignment) in 25 algae samples representing common morphological forms of *S. japonica*. All three gene regions (*rbc*LS, *COI*, and *ITS*) with comparable length were available in GenBank for some species related to *S. japonica*, which we have included in the analysis. The lengths of the GenBank concatenated sequences are (in parentheses): *S. japonica* (3,182 bp), *S. diabolica* (2,894 bp), *S. longipedalis* (2,896 bp), *S. ochotensis* (2,689 bp), *S. religiosa* (2,896 bp), *S. angustata* (4,256 bp), *S. coriacea* (2,711 bp), *S. latissima* (3,748 bp), and *L. digitata* (4,245 bp) (see Additional file 3 for GenBank accession numbers and full species names).

**Figure 1:**
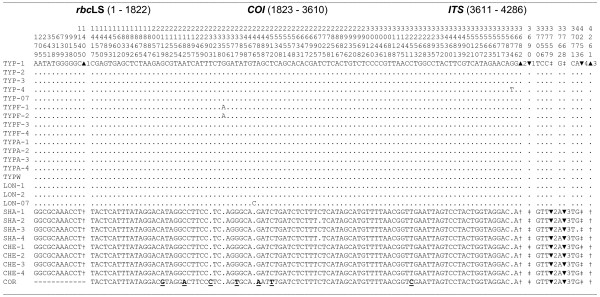
**Nucleotide substitution sites in the *rbc *LS, *COI *, and *ITS *gene regions of the *Saccharina japonica *morphological forms. ** The numbers above the top sequence represent the position of segregating sites and the start of a deletion or insertion (see also Additional file 4). Nucleotides are numbered from the beginning of our sequence. Dots indicate the same nucleotide as the reference sequence. The hyphens represent deleted nucleotides. ▴ denotes a deletion; † denotes the absence of a deletion; ▼ denotes an insertion; ‡ denotes the absence of an insertion. The sample abbreviations are in the section "Methods". COR: *S. coriacea*. The underlined nucleotides in bold face show fixed differences between COR and the other sequences.

Figure 1 shows 114 nucleotide substitutions detected in three gene regions (18 sites in *rbc*LS, 88 sites in *COI*, and 8 sites in *ITS*) among the 25 algae samples. In addition, there were seven length polymorphisms in the *rbc*LS intergenic spacer and *ITS* regions (Additional file 4); no length polymorphisms were found in the *COI* gene. All algae samples sharply split up in two groups. The first group includes all samples of the TYP (including TYPF, TYPA, and TYPW) and LON forms; the second group includes the SHA form and one unknown species from the Chernoruch'e bay (CHE). The genetic structure is completely congruent for all three gene regions studied (Figure 1). The two groups (denoted as “TYP” and “SHA” lineages) differ by 109 fixed single nucleotide differences, as well as by seven fixed length polymorphisms. The lineages are differentially associated with indels. The TYP lineage is completely associated with three inferred deletions (13-, 4-, and 8-bp, within the *rbc*LS intergenic spacer and *ITS* region; ▴1, ▴2, and ▴3, Figure 1) and two inferred insertions (▼1 and ▼4) within the *ITS* region. The SHA lineage is completely associated with two inferred 1-bp insertions within the *ITS* region (▼2 and ▼3). The difference between the TYP and SHA lineages based on the full concatenate (including the three gene regions, *rbc*LS, *COI*, and *ITS*) is highly significant (*F*_st_ = 0.9949, *P* < 0.00001); the total sequence divergence (*D*_xy_) between the lineages is 0.0261 (ignoring indels).

**Figure 2:**
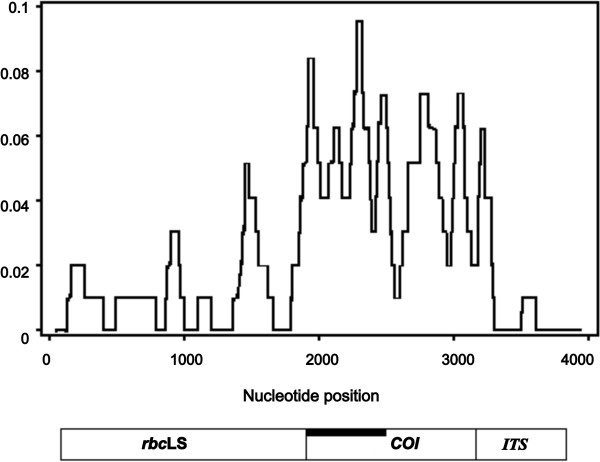
**Sliding window plot of nucleotide divergence for three gene regions (*rbc *LS, *COI *, and *ITS*) between the TYP and SHA forms of *Saccharina japonica. *** Window sizes are 100 nucleotides with one-nucleotide increments. A schematic representation of the regions is displayed at bottom. The thick black line marks the *COI* region investigated by McDevit and Saunders [43].

The three gene regions differed in levels of divergence between the TYP and SHA lineages (Figure 2). Total nucleotide divergence between TYP and SHA lineages is low for *rbc*LS (*D*_xy_ = 0.0099 ± 0.0025) and *ITS* (*D*_xy_ = 0.0106 ± 0.0037), but significantly higher (more than four times) for *COI* (*D*_xy_ = 0.0481 ± 0.0051). The same tendency is found for comparisons between all other laminarialean species pairs (data not shown). Thus the *COI* gene represents the most effective and sensitive gene marker of the three studied here for the comparison of close *Saccharina* species, which is in accordance with the data obtained for red algae and kelps (e.g., [55-57]).

The estimates of nucleotide diversity for all sequences of *S. japonica* and for the morphological forms or haplotype lineages separately are low. In the pooled sample, the total nucleotide diversity is similar for the TYP (π = 0.00011) and SHA (π = 0.00016) lineages (based on the three gene regions studied); the results are in accordance with literature data on intraspecific variability in *S. japonica*[58].

**Figure 3:**
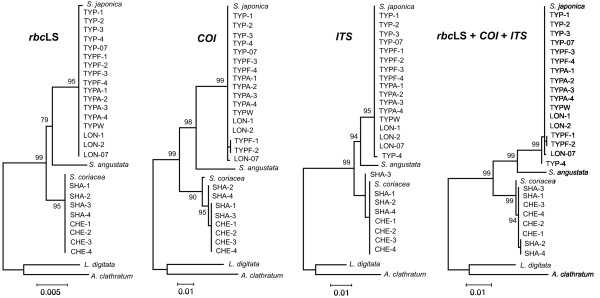
**Phylogenetic trees of the *rbc *LS, *COI, ITS *, and concatenate sequences (4,286 bp total) of*Saccharina japonica*morphological forms.** The topology of trees obtained with Maximum likelihood and Neighbor-joining methods were congruent. Numbers at the nodes are bootstrap percent support values based on 1,000 replications in Maximum likelihood analysis. *Saccharina latissima* was excluded from this analysis due to recombination in the *COI* gene (see text). Other comments as in Figure 1.

Figure 3 displays a maximum likelihood phylogram of the three gene regions separately and of the concatenate sequences obtained for the *S. japonica* forms in the present study, along with other *Saccharina* and *Laminaria* sequences obtained from GenBank. *S. ochotensis*, *S. religiosa*, *S. longipedalis*, and *S. diabolica* are conspesific with *S. japonica* (data not shown), as has been revealed previously [10,11]. All trees showed that the sequences from the TYP and LON forms form a single clade, with no evidence of discrete species heterogeneity (*F*_st_ = 0.0201; *P* = 0.6600; *K*_st_ = − 0.0099; *P* = 0.7480); total sequence divergence (*D*_xy_) is 0.0001. These data suggest that the TYP and LON morphological forms of *S. japonica* are not distinct biological species. In contrast, the sequences from the TYP and SHA lineages are distinct. The difference between the lineages is highly significant (*F*_st_ = 0.9949; *P* <0.0001; *K*_st_ = 0.8484; *P* <0.0001). The distance between them is 2.61% (2.77% including indels), which is in a range of the divergence between species observed within the genus *Saccharina*[1,41-43] suggesting that the TYP and SHA lineages would qualify as different species based on a phylogenetic species concept.

### Species identity

Comparison of sequences obtained during this study with those available in the GenBank database suggests that the TYP lineage represents *S. japonica*, whereas the SHA lineage is closely related to a group of *Saccharina* species that include *S. coriacea*, *S. cichorioides*, *S. sachalinensis*, and *S. yendoana* (the last three species are not represented in Figure 3 because the *COI* sequences are not available for them). The distance between the SHA lineage and this group of *Saccharina* species (represented by *S. coriacea* in Figures 1 and 3) is low and not significant (*F*_st_ = 0.9242; *P* = 0.3300; *K*_st_ = 0.3890; *P* = 0.3300); the *rbc*LS and *ITS* regions are identical but the *COI* gene shows seven single nucleotide differences (Figure 1). Total sequence divergence is 0.38% (for the full 4,286-bp concatenate alignment), which is below the species divergence level observed in algae [1,41] and other eukaryotes [59]. Yotsukura and colleagues [8,9,11,12] have revealed high genetic similarity between *S. coriacea*, *S. cichorioides*, *S. sachalinensis*, and *S. yendoana* and suggested their conspecificity, in accordance with earlier results of Petrov [6], who treated *L. coriacea* and *L. sachalinensis* as intraspecific forms of *L. cichorioides* (the status of *L. yendoana* was not discussed by Petrov [6].) Following the priority rule of the International Code of Botanical Nomenclature, Selivanova and colleagues [13] introduced a new nomenclature combination: *S. cichorioides* with four intraspecific forms. The conspecificity of *S. coriacea*, *S. cichorioides*, *S. sachalinensis*, and *S. yendoana* was suggested on the basis of low genetic divergence detected with ITS-1, ITS-2, RubisCO spacer, and *rbc*L sequences [8,9,11-13,60]. In the present work we have detected low divergence between the SHA lineage and the group of *Saccharina* species listed above using all three gene markers, including the *COI* gene (*D*_xy_ = 0.0050), which is considerably more informative than the *rbc*LS and *ITS* markers (see above). Consequently, we conclude that the SHA form belongs to the *S. cichorioides* group (all “species” representing the group were previously synonymized based on genetic data as pointed out) and it may represent a morphological ecotype adapted to supralittoral environments. *S. cichorioides* is more heat resistant than *S. japonica* and its main populations are more southward in the Sea of Japan. However, this morphologically and ecologically versatile species (as it follows from our data) is capable of occupying different environments and successfully compete with local species in the northern Primorye coast region under hyposalinity osmotic stress and increased water temperature (see Background).

The unknown *Saccharina* collected in the Bay Chernoruch'e (CHE) are genetically identical to the SHA form, even though they have substantial differences in external morphologies (T. N. Krupnova, unpublished observations) but occupy similar depths (0.5 m). It has been suggested that laminarialean algae from the supralittoral zone represent *S. angustata*[26]. However, this is not the case (see Figure 3): the SHA form is close to the *S. cichorioides* group (represented by *S. coriacea* in Figure 3) and clearly different from *S. angustata*. The distance between the SHA form and *S. angustata* is 2.63% (which is consistent with interspecific divergence level), indicating species taxonomical range for these two evolutionary lineages. Thus, *S. angustata* was not found among 25 algal samples collected from distant areas of the Northern Primorye region. This species is common in the Southern Kuril Islands and Japan islands [4,26], but it rarely occurs (if at all) along the continental coast of the Japan Sea.

### Non-uniform pattern of divergence in the *COI* gene

**Figure 4:**
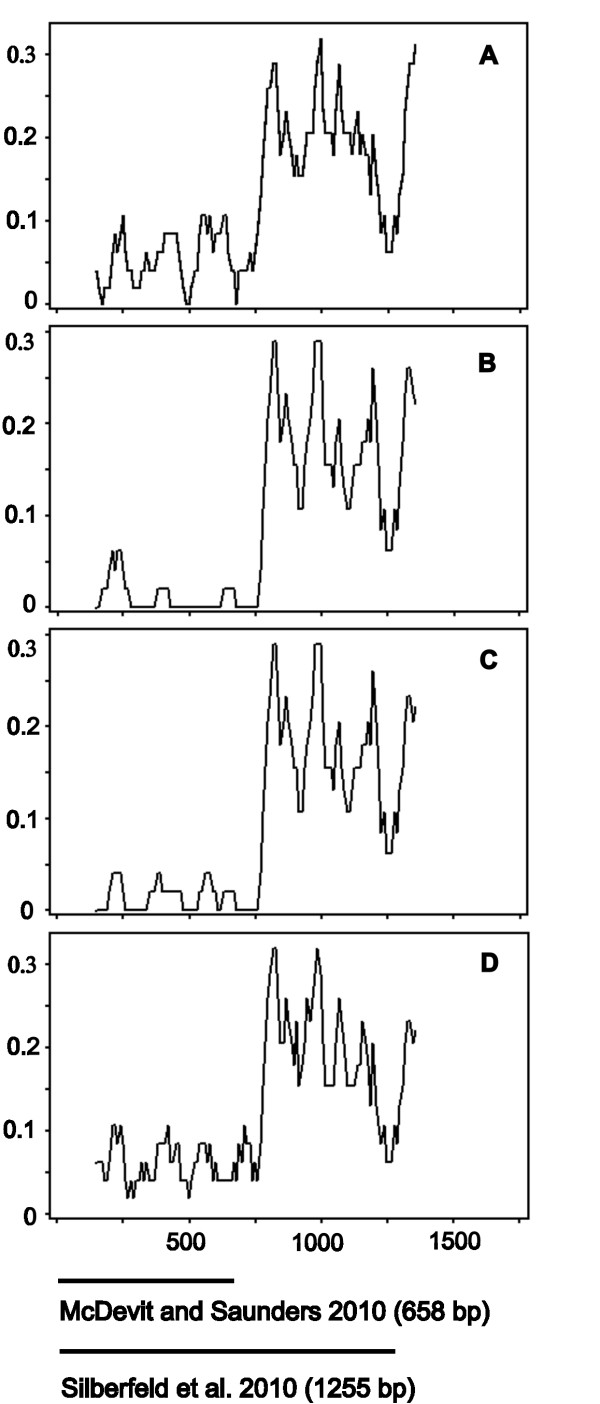
**Sliding-window plots of divergence along the *COI *gene region between *S. latissima *and other laminarialean algae: *S. japonica *(A), the SHA form (B), *S. coriacea *(C), and *S. angustata *(D). ** Window sizes are 50 nucleotides with ten-nucleotide increments. The thick black lines at the bottom mark the *COI* regions investigated by McDevit and Saunders [43] and Silberfeld with colleagues [2].

Full length of the *COI* coding region in laminarialean algae is around 1.6 kb (1,602 bp in *Saccharina*). The 5’ part of the gene (~ 650 bp in length; 5’-*COI*) has been chosen as a “barcoding” region [61] and recommended for algal species identification [41-43,61]. We have shown above that the most pronounced differences between *Saccharina* species are detected within the *COI* gene. However, the level of divergence is highly non-uniform within the *COI* gene in the comparisons, including *S. latissima* and other *Saccharina* species (Figure 4A – D). The average divergence for the 5’-*COI* is 0.0380 ± 0.0106 but 4.2 times more, 0.1579 ± 0.0034 for the 3’-*COI* (paired samples *t*-test *P* = 0.0001; Mann–Whitney test *P* = 0.0090; Table 1). This difference is also obvious for the comparisons with other laminarialean species for which long enough *COI* sequences are available in GenBank [2,11]: *Laminaria digitata*, *Alaria esculenta*, *Ecklonia radiata*, *Undaria pinnatifida*, and *Agarum clathratum*. The average divergence for the 5’-*COI* between these species is 0.1011 ± 0.0051 but 1.5 times more, 0.1516 ± 0.0030, for the 3’-*COI* (paired samples *t*-test *P* = 0.0022; Mann–Whitney test *P* = 0.0090 (Table 1). The pattern is the same for all species pair comparisons: the 5’-*COI* (~ 740 bp in length) was significantly less diverged between *S. latissima* and other laminarialean algae than the 3’-*COI* (~ 862 bp in length) (Figure 4A – D; Table 1).

**Table 1:** **Pairwise distances (with SE) between *Saccharina latissima *and close species, based on different segments of *COI ***

	**5’-*COI***	**3’-*COI***	**Full*COI***
Species pair	*D*_xy_ ± SE	*D*_xy_ ± SE	*D*_xy_ ± SE
*S. latissima* – *S. coriacea*	0.0141 ± 0.0049	0.1488 ± 0.0135	0.0774 ± 0.0081
*S. latissima* – SHA	0.0106 ± 0.0042	0.1508 ± 0.0136	0.0765 ± 0.0080
*S. latissima* – TYP	0.0528 ± 0.0091	0.1647 ± 0.0138	0.1054 ± 0.0093
*S. latissima* – *S. japonica*	0.0528 ± 0.0091	0.1647 ± 0.0138	0.1054 ± 0.0093
*S. latissima* – *S. angustata*	0.0599 ± 0.0100	0.1607 ± 0.0138	0.1073 ± 0.0094
*S. latissima* – *Laminaria digitata*	0.0915 ± 0.0115	0.1488 ± 0.0143	0.1185 ± 0.0101
*S. latissima* – *Agarum clathratum*	0.0951 ± 0.0115	0.1607 ± 0.0146	0.1259 ± 0.0101
*S. latissima* – *Ecklonia radiata*	0.1039 ± 0.0120	0.1548 ± 0.0140	0.1278 ± 0.0103
*S. latissima* – *Alaria esculenta*	0.0951 ± 0.0118	0.1508 ± 0.0137	0.1213 ± 0.0097
*S. latissima* – *Undaria pinnatifida*	0.1197 ± 0.0130	0.1429 ± 0.0142	0.1306 ± 0.0103
*S. latissima* – *Leathesia difformis*	0.1655 ± 0.0144	0.1290 ± 0.0134	0.1483 ± 0.0110
*S. latissima* – *Asperococcus bullosus*	0.1532 ± 0.0143	0.1349 ± 0.0138	0.1446 ± 0.0104
*S. latissima* – *Punctaria latifolia*	0.1602 ± 0.0143	0.1349 ± 0.0138	0.1483 ± 0.0110
*S. latissima* – *Elachista fucicola*	0.1989 ± 0.0166	0.1627 ± 0.0142	0.1819 ± 0.0115
*S. latissima* – *Hincksia granulosa*	0.1549 ± 0.0144	0.1290 ± 0.0131	0.1427 ± 0.0109
*S. latissima* – *Scytosiphon lomentaria*	0.1637 ± 0.0148	0.1349 ± 0.0136	0.1502 ± 0.0110
*S. latissima* – *Petalonia fascia*	0.1532 ± 0.0145	0.1468 ± 0.0137	0.1502 ± 0.0109
*S. latissima* – *Colpomenia peregrina*	0.1495 ± 0.0138	0.1468 ± 0.0144	0.1483 ± 0.0107
*S. latissima* – *Ectocarpus siliculosus*	0.1373 ± 0.0139	0.1627 ± 0.0143	0.1493 ± 0.0109
*S. latissima* – *Pylaiella littoralis*	0.1585 ± 0.0143	0.1607 ± 0.0144	0.1595 ± 0.0113

5’-*COI*: the 658-bp fragment covering the 5’-flanking region of the *COI* gene. The region starts 123 bp downstream of the *COI* start codon and ends 822 bp upstream of the *COI* stop codon. This fragment has been recommended for algae “barcoding” (species identification) [41-43,61]. 3’-*COI*: the 597-bp fragment covering the 3’-flanking region of the *COI* gene that starts 781 bp downstream of the *COI* start codon and ends 225 bp upstream of the *COI* stop codon. This fragment was not investigated by the above authors, but has been recently used (along with the 5’-*COI* region) for algae phylogenetic reconstruction [2]. Full *COI*: the 1378-bp fragment covering most of the *COI* gene. The fragment starts 123 bp downstream of the *COI* start codon and ends 225 bp upstream of the *COI* stop codon (the full *COI* region represents the largest sequence available for laminarialean algae in GenBank, excluding species for which full mtDNA sequences have been obtained). See Additional file 3 for the GenBank accession numbers.

The difference in the level of divergence between the *S. latissima* and other species for the 5’-*COI* and 3’-*COI* regions is highly pronounced (Figure 4; Table 1). The BLAST procedure (limited by the current GenBank submissions) reveals, as expected, very high identity between the 5’-*COI* region of *S. latissima* and other *Saccharina* species; however, the 3’-*COI* region of *S. latissima* has paradoxical similarity (but not complete identity) to a number of species belonging to the order *Ectocarpales*. Indeed, there is a noticeable decrease in the divergence for the 3’-*COI* region between *S. latissima* and the following *Ectocarpales* species: *Leathesia difformis*, *Asperococcus bullosus*, *Punctaria latifolia*, *Elachista fucicola*, *Hincksia granulosa*, *Scytosiphon lomentaria*, and *Petalonia fascia*. The average divergence for the 5’-*COI* between *S. latissima* and these species is 0.1642 ± 0.0061, but significantly less, 0.1389 ± 0.0046, for the 3’-*COI* (paired samples *t*-test *P* = 0.0007; Mann–Whitney test *P* = 0.0087; Table 1). Other *Ectocarpales* species for which long enough *COI* sequences are available (*Pylaiella littoralis*, *Ectocarpus siliculosus*, and *Colpomenia peregrina*) do not show any difference in the divergence level between 5’-*COI* and 3’-*COI* regions (Table 1).

### Phylogenetically discordant signals within the *COI* gene

**Figure 5:**
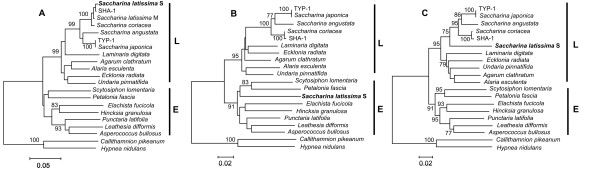
**Phylogenetic trees based on different fragments of the *COI *coding region: (A) 5’- *COI *, (B) 3’- *COI *, and (C) full *COI *region. ** Representative sequences of the orders *Laminariales* (L) and *Ectocarpales* (E) included in these trees are marked by vertical lines. Red algae *COI* sequences of *Callithamnion pikeanum* and *Hypnea nidulans* (GenBank accession numbers EU194965 and FJ694907, respectively) are used as outgroups. Note the changed position of *S. latissima* (in bold) depending on the *COI* region used for the tree. The *S. latissima COI* sequence denoted as “S” is from [2]. See Additional file 3 for GenBank accession numbers. Other comments as in Table 1 and Figure 3.

Thus, the difference in the level of divergence between the 5’-*COI* and 3’-*COI* regions is significant and exhibits an opposite pattern for comparisons between *S. latissima vs. Laminariales* species and *S. latissima vs. Ectocarpales* species: the 5’-*COI* region of *S. latissima* has obvious similarity to *Saccharina* species, whereas the 3’-*COI* regions has unexpected similarity with some *Ectocarpales* species. As a consequence, the position of *S. latissima* on 5’- and 3’-*COI* based trees are sharply different (Figure 5); on the 5’-*COI* based tree, *S. latissima* is within the same cluster with other *Saccharina* species but not distinguishable from *S. coriacea* (Figure 5A). On the 3’-*COI* tree *S. latissima* is significantly different from *Laminariales* algae and clusters with some species of the order *Ectocarpales* (Figure 5B). On the full length *COI* tree, *S. latissima* is within the order *Laminariales* (Figure 5C), but significantly different from other *Saccharina* species. The high similarity of the *S. latissima* 3’-*COI* region with *Ectocarpales* is surprising considering the large evolutionary distance between the two brown algae orders, which separated around 100 Ma [2].

### Recombination in the *COI* gene

Based on these observations we suspected that the unusual patterns of the *COI* gene divergence in comparisons of *S. latissima* with other species might reflect historical hybridization event(s) between representatives of distantly related *Ectocarpales* and *Laminariales* algae, which might have resulted in interspecific recombination of mitochondrial DNA. We therefore analyzed the *COI* alignments for evidence of recombination (and breakpoints) using various recombination detection methods implemented in the program RDP3 [62] (Table 2). The parental and recombinant sequences were determined using the VisRD method [63], modified version of PHYLPRO [64], and EEEP [65] also implemented in RDP3 (default settings).

**Table 2:** **Recombination assessed by six different methods (implemented in RDP3; see [ **[62]**])**

**Method**	**Reference**	**Recomb. species**	**Average*P* - value**
RDP	[66]	*S. latissima*	2.739 × 10^-12^
		*C. retorta*	3.167 × 10^-13^
GENECONV	[67]	*S. latissima*	3.815 × 10^-11^
		*C. retorta*	1.179× 10^-15^
BOOTSCAN	[68,69]	*S. latissima*	2.170 × 10^-06^
		*C. retorta*	1.416 × 10^-13^
MAXCHI	[70]	*S. latissima*	3.182 × 10^-10^
		*C. retorta*	5.361× 10^-08^
CHIMAERA	[71]	*S. latissima*	1.459 × 10^-08^
		*C. retorta*	4.560× 10^-11^
SISCAN	[72]	*S. latissima*	1.071 × 10^-23^
		*C. retorta*	4.285× 10^-25^

**Figure 6:**
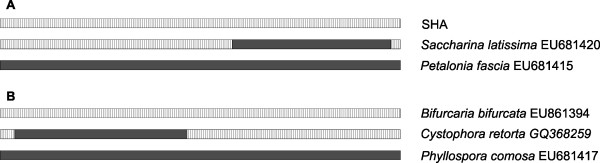
**Schematic representation of recombination events in the *COI *gene region from *Saccharina latissima *(A) and *Cystophora retorta *(B). ** The parental sequences are from *Petalonia fascia * and the SHA form (for *S. latissima *); and *Bifurcaria bifurcata * and *Phyllospora comosa * (for *C. retorta *).

All six methods detected a single recombination event in *S. latissima* within the 1,257 bp *COI* region (Figure 6A), with high statistical support (Table 2). The *COI* sequences from *Petalonia fascia* (*Ectocarpales*) and the SHA form (*Laminariales*) were major and minor parents, respectively. The recombination event involved the 3’-*COI* region, encompassing a 542 bp-long segment, between positions 1227 and 685.

Thus, the parental *COI* sequences of *S. latissima* come from different algae orders, *Ectocarpales* and *Laminariales*. The recombination would not have been detected if the alignment would be limited to the 5’-*COI* barcoding region. An interesting feature of algal evolution would not have been detected. Moreover, incorrect phylogenies would be obtained for species with historical hybridization events (see Figure 5).

To understand the uniqueness of this case of historical hybridization revealed in *S. latissima*, we analyzed additional, long enough *COI* sequences (~ 1200 bp; Additional file 3) of brown algae obtained from GenBank. We analyzed 57 sequences (GeneBank’s plus our own sequences) and detected one more highly significant instance of *COI* recombination between representatives of brown algae belonging to the families *Sargassacea* and *Seirococcaceae*. A recombinant *COI* sequence was revealed in *Cystophora retorta* (order *Fucales*, family *Sargassacea*). The *COI* sequences from *Phyllospora comosa* (order *Fucales*, family *Seirococcaceae*) and *Bifurcaria bifurcata* (order *Fucales*, family *Sargassacea*) were the major and minor parents, respectively. The recombination spanned the 5’-*COI* region (Figure 6B) with a beginning breakpoint at position 540 and ending breakpoint position 16. This recombination event involves representatives of two brown algae families, *Sargassacea* and *Seirococcaceae*, which diverged around 73 Ma [2]. Also, as in case of *S. latissima*, different and misleading phylogenetic patterns would come about depending on the *COI* region used in tree reconstruction (data not shown).

The recombination analysis has confirmed that the evolutionary histories of *S. latissima* and *C. retorta* have been influenced by gene flow between two highly diverged algae lineages, the orders *Ectocarpales* and *Laminariales* (for *S. latissima*) and between the families *Sargassacea* and *Seirococcaceae* (for *C. retorta*). The *COI* gene in *S. latissima* and *C. retorta* represents historical recombinant products between two distantly related ancestors. The level of *COI* divergence is 14.3% and 14.7% between the parental sequences of *C. retorta* and *S. latissima*, respectively, which seems very high for the occurrence of successful recombination (see, for instance, [73]). However, it has been shown [74] that recombination may occur among highly divergent maternal and paternal mtDNA sequences of the sea mussel *Mytilus galloprovincialis* that differ by > 20%.

There are multiple evidences of mtDNA recombination in plant, fungi, and animals, including human (reviews in [75-77]). Mitochondrial heteroplasmy (the presence of more than one mitochondrial genome in an individual) that could facilitate recombination has been reported in the brown alga *Fucus*[78,79]. Hybrid speciation is widespread in plants [80-82] and mosaic genomes due to hybrid speciation are common in many marine microorganisms, plants and animals (review in [83]). Interspecific hybridization has been detected in the brown algal genus *Fucus*[84-87] and the red algal genus *Porphyra*[88]. However, instances of interspecific mtDNA recombination were not, to our knowledge, previously known for algae; it is an infrequent phenomenon detected in fish and primates [89,90].

The two examples of interspecific recombination come from published work by Silberfeld with colleagues [2]. It might be suggested that these recombinations are artificial, resulting from PCR errors. However, the architecture of the recombinant sequences is not a mosaic composition from different parts of the parental sequences; the recombinants are similar but not identical to any other sequence used in this study. These observations suggest that PCR errors are unlikely to account for these recombinant sequences. Nevertheless, more sampling will be necessary in order to settle this issue of putative hybrid speciation in algae.

Our results are relevant concerning the DNA barcoding for algae and possibly other organisms. The 5’-*COI* “barcode” region is not representative and might be even misleading (at least in case of *S. latissima* and *C. retorta*) in resolving taxonomic relationships between algal species. Although 5’-*COI* barcoding is practically convenient (because 600–700 bp can readily be sequenced in a single run); it may not be suitable.

## Discussion

We have found that the TYP and LON morphological forms of *Saccharina japonica* are genetically very similar and, therefore, they might not be thought of as distinct biological species, as suggested by Gusarova and Ivanova [17]. The SHA form, however, exhibits distinctive genetic differences from the TYP and LON forms (2.6% total DNA divergence) and it is closely related to the *S. cichorioides* species group. The SHA form might be thought of as a morphological variety of *S. cichorioides* that inhabits an ecological niche – the supralittoral zone – new for this species. Our results are of practical consequence. Zoospores of the SHA form are used to replenish populations of the TYP form suffering from overharvesting [19]. This practice is inappropriate because the TYP and SHA forms represent different species. Genetic analysis can, indeed, help to select appropriate stocks for mariculture.

Thus, the genetic data suggest the existence of two close *Saccharina* lineages inhabiting the Primorye coastal region, *S. japonica* (TYP + LON) and *S. cichorioides* (SHA). Significant genetic differences between the SHA form and the TYP plus LON forms are not surprising: these two lineages are also highly different in morphology and ecological preferences. Less expected is the nearly complete genetic identity of the TYP and LON forms, because these forms have drastic differences in morphology, life history traits, and ecology. Taking into account that interbreeding between the LON and TYP forms is highly unlikely (see Background), and also the fact that phenotypic plasticity may have potential roles for adaptive evolution and speciation, as it has been shown for many species, including algae (e.g., [27-31]), we propose that the TYP and LON forms represent important resources for algae diversification that enable successful adaptation to contrasting shallow- and deep-water marine environments. The present results for *Saccharina* are in accordance with our previous results concerning two morphological forms of the sea urchin *Strongylocentrotus intermedius*, which are clearly different morphologically but very similar genetically [91].

Transplant site experiments are not likely to be informative concerning the taxonomical status of the *Saccharina* morphological forms since both, the LON form, which is genetically similar to the TYP form, and the SHA form, which is genetically quite different from the TYP form, exhibit drastic morphological transformations when transplanted to the TYP habitat area, so that they become morphologically indistinguishable from the TYP form [23,24]. Breeding experiments would not likely be informative either to resolve the taxonomic status of the TYP and LON forms, because even distantly related algal species produce highly viable first generation hybrids [88,92-95]. Indeed, the biological species concept is not particularly helpful in kelps [92,96]. Kraan and Guiry [92] have, for example, shown that interspecific sequence divergence in *Alaria* is smaller than intraspecific sequence divergence. That is, there is more variation within a species than between two species of the same genus, which casts doubts on the morphological and biological species concepts used in *Alaria* and on the usefulness of hybridization studies in assessing species-level differences [97-100]. Conflict between speciation decisions based on morphological or molecular characteristics comes about due to the drastically different rates at which molecular and morphological changes accumulate [100]. The use of a morphological and biological species concept to separate *Alaria* or *Saccharina* species is not satisfactory and does not fully reflect phylogenetic relationships.

The data show that there is no consistent relationship between morphological and genetic variation in the algae *Saccharina* (present data) and *Alaria*[96], as well as in sea urchins [91]. The results of Druehl and Saunders [96] and Kraan and Guiry [92] demonstrate that fertility barriers, which in most cases indicate complete reproductive isolation, may arise without affecting genetic divergence in the particular genes under investigation [101]. Our results support a relatively minor contribution of genetic factors to the TYP and LON morphological forms differences observed in the three gene markers, suggesting an important phenotypic plasticity basis for the variability in morphology, life history, and ecology in *S. japonica*.

The relative importance of genes and phenotypic plasticity in the generation of adaptive variation is a far from resolved issue [102]. The possibility that plasticity could play a major role, however, has recently become more commonly considered than it was traditionally believed [103-105]. A body of evidence suggests that plasticity may promote adaptive divergence in various systems, often followed by genetic changes in the direction of the plastic response [106] (see however, [107]). Furthermore, phenotypic plasticity enhances the survival and reproductive success of individuals by contributing to their ability to cope with environmental changes. In this way, it enables potential adaptation to new niches [108], and therefore can promote important biological processes such as adaptation, divergence, reproductive isolation, and evolutionary innovation [28,109,110]. Environmentally induced phenotypic variation has been argued to be a more likely source of evolutionary novelty than new mutations [103,104]. It has, indeed, been asserted that phenotypic plasticity can accelerate adaptive evolution by shortening the time a population needs to discover a new genotypic network [111] even in the case of recurrent rapid changes in gene regulation [112].

A number of experimental studies have revealed the mechanisms of phenotypic plasticity using an integrative biology approach (review in [113]). The overall results indicate that phenotypic plasticity has an important role in adaptive evolution. This consideration, in turn, points out the importance of characterizing diverging phenotypes and identifying relevant evolutionary forces acting on those phenotypes. A great number of intraspecific morphological varieties are known for laminarialean algae (review in [14]). Further studies of algae morphological forms are important and necessary to further understand how biological diversity is generated and maintained in evolution.

## Conclusions

1. We have investigated the genetic make-up in three *S. japonica* forms, TYP, LON, and SHA. We have found that in spite of their drastic differences in morphology, life history and ecology, the TYP and LON forms are genetically indistinguishable and clearly different from the SHA form, which has a close genetic relationship with *S. cichorioides*. Taking into account the potential evolutionary significance of phenotypic plasticity, the TYP and LON forms may represent an important resource for algal evolutionary diversification and successful adaptation to contrasting marine environments.

2. The three gene markers used in the present work have different sensitivity for algal species identification; the mitochondrial *COI* gene is the most efficient gene marker. However, we found that the designated 5’-*COI* “barcoding” region is not enough and may even be misleading for purposes of identification, at least in comparisons that include species with evolutionary histories involving gene flow between distantly related algae lineages.

3. Populations of the TYP and LON forms are depressed owing to overharvesting; restriction programs are in play to enhance these populations. But the correct foundation stocks must be used. Zoospores of the SHA form should not be used to seed the fields of the TYP and LON forms because they belong to different evolutionary lineages. Genetic determination should be used to select correct foundation stocks in mariculture and management programs.

## Competing interests

The authors declare that they have no competing interests.

## Authors' contributions

ESB designed the study, carried out the molecular genetic studies, performed the sequence assembling and alignment, statistical analysis, and drafted the manuscript. TNK participated in the design of the study and collected algae samples. FJA participated in the design of the study and contributed to write the manuscript. All three authors read and approved the final manuscript.

## Note added in proof

Since the work described in this paper was completed and submitted for publication, sequences of the *COI* gene (most sensitive gene marker; see text) were obtained for typical individuals of *Saccharina cichorioides*. We detected no differences between the SHA form and *S. cichorioides*, which supports our conclusion about the conspecificity of the SHA form and *S. cichorioides*. The *COI* sequences have been deposited in GenBank under accession numbers JQ792007-JQ792010.

## Supplementary Material

Additional file 1 **Morphological, ecological, and life history traits differences between the morphological forms of *Saccharina japonica ***[15-17,20,26,114]. Click here for file

Additional file 2 PCR conditions. Click here for file

Additional file 3 **Alphabetical list of brown algal species with GenBank accession numbers of the nucleotide sequences used in this study**[2,43]. Click here for file

Additional file 4 **Coordinates of indels in the *rbc *LS and *ITS *gene regions of *Saccharina japonica *morphological forms. ** Click here for file
