# Supplementary material for: DNA variation in the phenotypically-diverse brown alga Saccharina japonica
Source: BMC Plant Biol. 2012 Jul 11;12:108. doi: 10.1186/1471-2229-12-108 (PMC3490969; doi:10.1186/1471-2229-12-108)
Supplement: Additional file 1 — Morphological, ecological, and life history traits differences between the morphological forms of Saccharina japonica [15-17,20,26,114]. [file 1471-2229-12-108-S1.doc]

**Additional file 1.** Morphological, ecological, and life history traits differences between the morphological forms of *Saccharina japonica*

|  | TYP (typical form) | LON (“longipes” form) | SHA (shallow-water form) | *S. cichorioides* |
| --- | --- | --- | --- | --- |
| Thallus color | Olive | Light olive | Dark olive | Olive |
| Thallus length | 2.0 – 3.5 m | 6.0 – 8.0 m (max. 12.0 m) | 0.3 – 1.5 m (max. 2.0 m) | 1.0 – 2.0 (max. 2.5 m) |
| Thallus width | 0.20 – 0.35 m | 0.45 – 0.50 m | 0.05 – 0.12 m | 0.10 – 0.30 m |
| Thallus thickness | 0.4 – 0.6 cm | 0.6 – 1.0 cm | 0.4 – 0.8 cm | 0.4 – 0.6 cm |
| Middle line | 0.10 – 0.18 m | 0.25 m – 0.30 m | There is no middle line or it occupies whole thallus width. | 0.08 – 0.20 m; There are bubbles along both sides of middle space in young individuals. |
| Total weight | 0.6 – 0.8 kg | 4.5 – 5.7 kg | 0.05 – 0.2 kg | 0.3 – 1.2 kg |
| Rhizoid | Short, thick, fascicled | Long, thin, branched | Short, thick, fascicled | Short, hick, fascicled |
| Cauloid | Roundish, short (up to 12 cm), thick | Flattish, long (up to 42 cm), sporadically turbinal | Very short (up to 2 cm), thick | Short (up to 5 cm) |

Additional file 1 (continued).

|  | TYP (typical form) | LON (“longipes” form) | SHA (shallow-water form) | *S. cichorioides* |
| --- | --- | --- | --- | --- |
| Thallus anatomy (histology): mucous ducts | Mucous ducts are located near the border with (or in) parenchyma | Mucous ducts are located in the cortex layer | Mucous ducts are located in the cortex layer | Mucous ducts are located in the cortex layer |
| Thallus anatomy (histology): cortex layer | Cortex layer consists of 5 – 8 rows of cell (3.3 – 3.9% of thallus thickness) | Cortex layer consists of 2 – 3 rows of cell (1.6 – 1.8% of thallus thickness) | Cortex layer consists of 8 – 10 rows of cell (50 – 60% of thallus thickness) | Cortex layer consists of 3 – 5 rows of cell (50 – 60% of thallus thickness) |
| Sporangia disposition on thallus | Sporangia are formed starting from the upper part of thallus | Sporangia are formed starting from the lower and upper part of thallus | Sporangia are formed starting from the upper part of thallus | Sporangia are formed starting from the upper part of thallus |
| Sporulation period | July – October (up to November in Northern Primorye coast region) | October – December | July – October | July – October |

Additional file 1 (continued).

|  | TYP (typical form) | LON (“longipes” form) | SHA (shallow-water form) | *S. cichorioides* |
| --- | --- | --- | --- | --- |
| Seedling | March – April (1 – 2 ^o^C) | July – August (4 – 10^o^C) | March – April (1 – 2 ^o^C) | March – April (1 – 2 ^o^C) |
| Depth | 5 – 11 m (max. 25 m) | 12 – 14 m (max. 28 m) | 0.1 – 0.5 m (max. 1 m) | 2 – 8 m (max. 12 m) |
| Distribution pattern and preferred bottom | Wide distribution over all species areas (warm-temperate Far Eastern waters); it grows along shore (coast-wisely); rocky bottom; exposed coast; open entrance capes | Restricted distribution (middle Primorye coastal region, the Sea of Japan and western Sakhalin, the Sea of Okhotsk); it grows at significant distance (300 – 1000 m) from sea-shore; gravel bottom | Wide distribution in the Primorye coast region, the Sea of Japan, including the Peter the Great Bay; gravel bottom | Wide distribution in the Primorye coast region, the Sea of Japan, including the Peter the Great Bay; gravel bottom |

The data are combined from [1-6]. Geographical distribution patterns data are available for the territory of the Russian Federation only.

**References for the Additional file 1**

1. Gail GI: **Laminariaceous algae of the Far Eastern seas.** Vestnik DV FAN SSSR 1936, **19**:31-64.

2. Sukhoveeva MV: **The algae distribution of along the Primorye coast region.** *Izv TINRO* 1967, **61**:255-260.

3. Petrov YuE, Sukhoveeva MV: ***Laminaria angustata* Kjellm. at the coasts of Primorskii region.** *Novit Syst Plant Non Vascularium* 1972, **9**:44-47.

4. Paimeeva LG: **Distribution and growth of *Laminaria japonica* Aresch f. *longipes* (Miyabe et Tokida) Petr. in northern Primorye.** In: *Commercial Algae and Their Use.* Moscow: Nauka; 1987:26-33.

5. Krupnova TN: **Development of sporogenous tissue in *Laminaria japonica* under the influence of changing environment.** *Izv TINRO* 2002, **130**:474-482.

6. Gusarova IS, Ivanova NV: **Intraspecific systematic of *Laminaria japonica* at the continental coast of the Japan Sea.** *Izv TINRO* 2006, **147**:157-168.
