# Supplementary material for: DNA variation in the phenotypically-diverse brown alga Saccharina japonica
Source: BMC Plant Biol. 2012 Jul 11;12:108. doi: 10.1186/1471-2229-12-108 (PMC3490969; doi:10.1186/1471-2229-12-108)
Supplement: Additional file 2 — PCR conditions. [file 1471-2229-12-108-S2.doc]

**Additional file 2.** PCR conditions.

The PCR reactions were carried out in final volumes of 25 µl using TaKaRa Ex Taq™ in accordance with the manufacturer’s description (Takara Biotechnology Co., Ltd.). The PCR reaction mixtures were placed in a DNA thermal cycler (Eppendorf, Mastercycler Gradient), incubated 5 min at 95° and subjected to 32 cycles of denaturation, annealing, and extension: 95° for 30 sec, 52° for 30 sec, and 72° for 1.0 min, with a final 5-min extension period at 72° for the *COI* gene; 95° for 30 sec, 53° for 30 sec, and 72° for 1.5 min, with a final 7-min extension period at 72° for the *rbc*LS gene; 95° for 30 sec, 53° for 30 sec, and 72° for 1.5 min, with a final 7-min extension period at 72° for the rDNA (*ITS*) region. The sequences of both strands were determined, using overlapping internal primers spaced, on average, 500 nucleotides. At least two independent PCR amplifications were sequenced in both directions to correct for possible cloning or sequencing errors.
