# Supplementary material for: DNA variation in the phenotypically-diverse brown alga Saccharina japonica
Source: BMC Plant Biol. 2012 Jul 11;12:108. doi: 10.1186/1471-2229-12-108 (PMC3490969; doi:10.1186/1471-2229-12-108)
Supplement: Additional file 3 — Alphabetical list of brown algal species with GenBank accession numbers of the nucleotide sequences used in this study[2,43]. [file 1471-2229-12-108-S3.doc]

**Additional file 3.** Alphabetical list of brown algae species with GenBank accession numbers of the nucleotide sequences used n this study. Missing sequences are designed by a dash.

| Taxon | *COI* | *rbc*LS | *ITS* |
| --- | --- | --- | --- |
| *Agarum clathratum* | GQ368254 | AY851542 | FJ042754 |
| *Alaria esculenta* | [EU681388](http://www.sciencedirect.com/science?_ob=RedirectURL&_method=externObjLink&_locator=ncbi-n&_origin=article&_zone=art_page&_plusSign=%2B&_targetURL=http%253A%252F%252Fwww.ncbi.nlm.nih.gov%252Fentrez%252Fquery.fcgi%253Fcmd%253Dsearch%2526db%253Dnucleotide%2526doptcmdl%253Dgenbank%2526term%253DEU681388) | - | - |
| *Analipus japonicus* | [EU681389](http://www.sciencedirect.com/science?_ob=RedirectURL&_method=externObjLink&_locator=ncbi-n&_origin=article&_zone=art_page&_plusSign=%2B&_targetURL=http%253A%252F%252Fwww.ncbi.nlm.nih.gov%252Fentrez%252Fquery.fcgi%253Fcmd%253Dsearch%2526db%253Dnucleotide%2526doptcmdl%253Dgenbank%2526term%253DEU681389) | - | - |
| *Ascophyllum nodosum* | [EU681390](http://www.sciencedirect.com/science?_ob=RedirectURL&_method=externObjLink&_locator=ncbi-n&_origin=article&_zone=art_page&_plusSign=%2B&_targetURL=http%253A%252F%252Fwww.ncbi.nlm.nih.gov%252Fentrez%252Fquery.fcgi%253Fcmd%253Dsearch%2526db%253Dnucleotide%2526doptcmdl%253Dgenbank%2526term%253DEU681390) | - | - |
| *Ascoseira mirabilis* | [EU681391](http://www.sciencedirect.com/science?_ob=RedirectURL&_method=externObjLink&_locator=ncbi-n&_origin=article&_zone=art_page&_plusSign=%2B&_targetURL=http%253A%252F%252Fwww.ncbi.nlm.nih.gov%252Fentrez%252Fquery.fcgi%253Fcmd%253Dsearch%2526db%253Dnucleotide%2526doptcmdl%253Dgenbank%2526term%253DEU681391) | - | - |
| *Asperococcus bullosus* | [EU681392](http://www.sciencedirect.com/science?_ob=RedirectURL&_method=externObjLink&_locator=ncbi-n&_origin=article&_zone=art_page&_plusSign=%2B&_targetURL=http%253A%252F%252Fwww.ncbi.nlm.nih.gov%252Fentrez%252Fquery.fcgi%253Fcmd%253Dsearch%2526db%253Dnucleotide%2526doptcmdl%253Dgenbank%2526term%253DEU681392) | - | - |
| *Bachelotia antillarum* | [EU681393](http://www.sciencedirect.com/science?_ob=RedirectURL&_method=externObjLink&_locator=ncbi-n&_origin=article&_zone=art_page&_plusSign=%2B&_targetURL=http%253A%252F%252Fwww.ncbi.nlm.nih.gov%252Fentrez%252Fquery.fcgi%253Fcmd%253Dsearch%2526db%253Dnucleotide%2526doptcmdl%253Dgenbank%2526term%253DEU681393) | - | - |
| *Bifurcaria bifurcata* | [EU681394](http://www.sciencedirect.com/science?_ob=RedirectURL&_method=externObjLink&_locator=ncbi-n&_origin=article&_zone=art_page&_plusSign=%2B&_targetURL=http%253A%252F%252Fwww.ncbi.nlm.nih.gov%252Fentrez%252Fquery.fcgi%253Fcmd%253Dsearch%2526db%253Dnucleotide%2526doptcmdl%253Dgenbank%2526term%253DEU681394) | - | - |
| *Colpomenia peregrina* | [EU681397](http://www.sciencedirect.com/science?_ob=RedirectURL&_method=externObjLink&_locator=ncbi-n&_origin=article&_zone=art_page&_plusSign=%2B&_targetURL=http%253A%252F%252Fwww.ncbi.nlm.nih.gov%252Fentrez%252Fquery.fcgi%253Fcmd%253Dsearch%2526db%253Dnucleotide%2526doptcmdl%253Dgenbank%2526term%253DEU681397) | - | - |
| *Cystophora retorta* | GQ368259 | - | - |
| *Cystoseira baccata* | [EU681399](http://www.sciencedirect.com/science?_ob=RedirectURL&_method=externObjLink&_locator=ncbi-n&_origin=article&_zone=art_page&_plusSign=%2B&_targetURL=http%253A%252F%252Fwww.ncbi.nlm.nih.gov%252Fentrez%252Fquery.fcgi%253Fcmd%253Dsearch%2526db%253Dnucleotide%2526doptcmdl%253Dgenbank%2526term%253DEU681399) | - | - |
| *Cystoseira nodicaulis* | [EU681400](http://www.sciencedirect.com/science?_ob=RedirectURL&_method=externObjLink&_locator=ncbi-n&_origin=article&_zone=art_page&_plusSign=%2B&_targetURL=http%253A%252F%252Fwww.ncbi.nlm.nih.gov%252Fentrez%252Fquery.fcgi%253Fcmd%253Dsearch%2526db%253Dnucleotide%2526doptcmdl%253Dgenbank%2526term%253DEU681400) | - | - |
| *Cystoseira tamariscifolia* | [EU681401](http://www.sciencedirect.com/science?_ob=RedirectURL&_method=externObjLink&_locator=ncbi-n&_origin=article&_zone=art_page&_plusSign=%2B&_targetURL=http%253A%252F%252Fwww.ncbi.nlm.nih.gov%252Fentrez%252Fquery.fcgi%253Fcmd%253Dsearch%2526db%253Dnucleotide%2526doptcmdl%253Dgenbank%2526term%253DEU681401) | - | - |
| *Desmarestia aculeata* | [EU681402](http://www.sciencedirect.com/science?_ob=RedirectURL&_method=externObjLink&_locator=ncbi-n&_origin=article&_zone=art_page&_plusSign=%2B&_targetURL=http%253A%252F%252Fwww.ncbi.nlm.nih.gov%252Fentrez%252Fquery.fcgi%253Fcmd%253Dsearch%2526db%253Dnucleotide%2526doptcmdl%253Dgenbank%2526term%253DEU681402) | - | - |
| *Desmarestia viridis* | [AY500367](http://www.sciencedirect.com/science?_ob=RedirectURL&_method=externObjLink&_locator=ncbi-n&_origin=article&_zone=art_page&_plusSign=%2B&_targetURL=http%253A%252F%252Fwww.ncbi.nlm.nih.gov%252Fentrez%252Fquery.fcgi%253Fcmd%253Dsearch%2526db%253Dnucleotide%2526doptcmdl%253Dgenbank%2526term%253DAY500367) | - | - |
| *Dictyota dichotoma* | [AY500368](http://www.sciencedirect.com/science?_ob=RedirectURL&_method=externObjLink&_locator=ncbi-n&_origin=article&_zone=art_page&_plusSign=%2B&_targetURL=http%253A%252F%252Fwww.ncbi.nlm.nih.gov%252Fentrez%252Fquery.fcgi%253Fcmd%253Dsearch%2526db%253Dnucleotide%2526doptcmdl%253Dgenbank%2526term%253DAY500368) | - | - |
| *Durvillaea potatorum* | [EU681405](http://www.sciencedirect.com/science?_ob=RedirectURL&_method=externObjLink&_locator=ncbi-n&_origin=article&_zone=art_page&_plusSign=%2B&_targetURL=http%253A%252F%252Fwww.ncbi.nlm.nih.gov%252Fentrez%252Fquery.fcgi%253Fcmd%253Dsearch%2526db%253Dnucleotide%2526doptcmdl%253Dgenbank%2526term%253DEU681405) | - | - |
| *Ecklonia radiata* | GQ368261 | - | - |
| *Ectocarpus* sp. | [EU681406](http://www.sciencedirect.com/science?_ob=RedirectURL&_method=externObjLink&_locator=ncbi-n&_origin=article&_zone=art_page&_plusSign=%2B&_targetURL=http%253A%252F%252Fwww.ncbi.nlm.nih.gov%252Fentrez%252Fquery.fcgi%253Fcmd%253Dsearch%2526db%253Dnucleotide%2526doptcmdl%253Dgenbank%2526term%253DEU681406) | - | - |
| *Elachista fucicola* | [EU681407](http://www.sciencedirect.com/science?_ob=RedirectURL&_method=externObjLink&_locator=ncbi-n&_origin=article&_zone=art_page&_plusSign=%2B&_targetURL=http%253A%252F%252Fwww.ncbi.nlm.nih.gov%252Fentrez%252Fquery.fcgi%253Fcmd%253Dsearch%2526db%253Dnucleotide%2526doptcmdl%253Dgenbank%2526term%253DEU681407) | - | - |
| *Fucus vesiculosus* | [AY494079](http://www.sciencedirect.com/science?_ob=RedirectURL&_method=externObjLink&_locator=ncbi-n&_origin=article&_zone=art_page&_plusSign=%2B&_targetURL=http%253A%252F%252Fwww.ncbi.nlm.nih.gov%252Fentrez%252Fquery.fcgi%253Fcmd%253Dsearch%2526db%253Dnucleotide%2526doptcmdl%253Dgenbank%2526term%253DAY494079) | - | - |
| *Halidrys siliquosa* | [EU681408](http://www.sciencedirect.com/science?_ob=RedirectURL&_method=externObjLink&_locator=ncbi-n&_origin=article&_zone=art_page&_plusSign=%2B&_targetURL=http%253A%252F%252Fwww.ncbi.nlm.nih.gov%252Fentrez%252Fquery.fcgi%253Fcmd%253Dsearch%2526db%253Dnucleotide%2526doptcmdl%253Dgenbank%2526term%253DEU681408) | - | - |
| *Himantothallus grandifolius* | GQ368262 | - | - |
| *Hincksia granulosa* | [EU681410](http://www.sciencedirect.com/science?_ob=RedirectURL&_method=externObjLink&_locator=ncbi-n&_origin=article&_zone=art_page&_plusSign=%2B&_targetURL=http%253A%252F%252Fwww.ncbi.nlm.nih.gov%252Fentrez%252Fquery.fcgi%253Fcmd%253Dsearch%2526db%253Dnucleotide%2526doptcmdl%253Dgenbank%2526term%253DEU681410) | - | - |
| *Hormosira banksii* | [EU681411](http://www.sciencedirect.com/science?_ob=RedirectURL&_method=externObjLink&_locator=ncbi-n&_origin=article&_zone=art_page&_plusSign=%2B&_targetURL=http%253A%252F%252Fwww.ncbi.nlm.nih.gov%252Fentrez%252Fquery.fcgi%253Fcmd%253Dsearch%2526db%253Dnucleotide%2526doptcmdl%253Dgenbank%2526term%253DEU681411) | - | - |
| *Laminaria digitata* | [AJ344328](http://www.sciencedirect.com/science?_ob=RedirectURL&_method=externObjLink&_locator=ncbi-n&_origin=article&_zone=art_page&_plusSign=%2B&_targetURL=http%253A%252F%252Fwww.ncbi.nlm.nih.gov%252Fentrez%252Fquery.fcgi%253Fcmd%253Dsearch%2526db%253Dnucleotide%2526doptcmdl%253Dgenbank%2526term%253DAJ344328) | AY851559 | AF319014 |
| *Leathesia difformis* | [EU681412](http://www.sciencedirect.com/science?_ob=RedirectURL&_method=externObjLink&_locator=ncbi-n&_origin=article&_zone=art_page&_plusSign=%2B&_targetURL=http%253A%252F%252Fwww.ncbi.nlm.nih.gov%252Fentrez%252Fquery.fcgi%253Fcmd%253Dsearch%2526db%253Dnucleotide%2526doptcmdl%253Dgenbank%2526term%253DEU681412) | - | - |
| *Notheia anomala* | [EU681413](http://www.sciencedirect.com/science?_ob=RedirectURL&_method=externObjLink&_locator=ncbi-n&_origin=article&_zone=art_page&_plusSign=%2B&_targetURL=http%253A%252F%252Fwww.ncbi.nlm.nih.gov%252Fentrez%252Fquery.fcgi%253Fcmd%253Dsearch%2526db%253Dnucleotide%2526doptcmdl%253Dgenbank%2526term%253DEU681413) | - | - |
| *Petalonia fascia* | [EU681415](http://www.sciencedirect.com/science?_ob=RedirectURL&_method=externObjLink&_locator=ncbi-n&_origin=article&_zone=art_page&_plusSign=%2B&_targetURL=http%253A%252F%252Fwww.ncbi.nlm.nih.gov%252Fentrez%252Fquery.fcgi%253Fcmd%253Dsearch%2526db%253Dnucleotide%2526doptcmdl%253Dgenbank%2526term%253DEU681415) | - | - |
| *Phyllariopsis brevipes* | GQ368264 | - | - |
| *Phyllospora comosa* | [EU681417](http://www.sciencedirect.com/science?_ob=RedirectURL&_method=externObjLink&_locator=ncbi-n&_origin=article&_zone=art_page&_plusSign=%2B&_targetURL=http%253A%252F%252Fwww.ncbi.nlm.nih.gov%252Fentrez%252Fquery.fcgi%253Fcmd%253Dsearch%2526db%253Dnucleotide%2526doptcmdl%253Dgenbank%2526term%253DEU681417) | - | - |
| *Punctaria latifolia* | [EU681418](http://www.sciencedirect.com/science?_ob=RedirectURL&_method=externObjLink&_locator=ncbi-n&_origin=article&_zone=art_page&_plusSign=%2B&_targetURL=http%253A%252F%252Fwww.ncbi.nlm.nih.gov%252Fentrez%252Fquery.fcgi%253Fcmd%253Dsearch%2526db%253Dnucleotide%2526doptcmdl%253Dgenbank%2526term%253DEU681418) | - | - |
| *Pylaiella littoralis* | [AJ277126](http://www.sciencedirect.com/science?_ob=RedirectURL&_method=externObjLink&_locator=ncbi-n&_origin=article&_zone=art_page&_plusSign=%2B&_targetURL=http%253A%252F%252Fwww.ncbi.nlm.nih.gov%252Fentrez%252Fquery.fcgi%253Fcmd%253Dsearch%2526db%253Dnucleotide%2526doptcmdl%253Dgenbank%2526term%253DAJ277126) | - | - |
| *Ralfsia fungiformis* | [EU681419](http://www.sciencedirect.com/science?_ob=RedirectURL&_method=externObjLink&_locator=ncbi-n&_origin=article&_zone=art_page&_plusSign=%2B&_targetURL=http%253A%252F%252Fwww.ncbi.nlm.nih.gov%252Fentrez%252Fquery.fcgi%253Fcmd%253Dsearch%2526db%253Dnucleotide%2526doptcmdl%253Dgenbank%2526term%253DEU681419) | - | - |
| *Saccharina angustata* | NC_013473 | AY851554 | AY465868 |
| *Saccharina coriacea* | NC_013475 | AB480850 | AB022803 |
| *Saccharina diabolica* | NC_013482 | AF318975 | AB022796 |
| *Saccharina japonica* | NC_013476 | DQ143101 | DQ143056 |
| *Saccharina latissima* | GU097789^a^ | AY851561 | AF319019 |
| *Saccharina latissima* | [EU681420](http://www.sciencedirect.com/science?_ob=RedirectURL&_method=externObjLink&_locator=ncbi-n&_origin=article&_zone=art_page&_plusSign=%2B&_targetURL=http%253A%252F%252Fwww.ncbi.nlm.nih.gov%252Fentrez%252Fquery.fcgi%253Fcmd%253Dsearch%2526db%253Dnucleotide%2526doptcmdl%253Dgenbank%2526term%253DEU681420)^b^ | AY851561 | AF319019 |
| *Saccharina longipedalis* | NC_013484 | AF318977 | AB022798 |
| *Saccharina ochotensis* | NC_013478 | AB428863 | AB428862 |
| *Saccharina religiosa* | NC_013477 | AF318979 | AB428850 |
| *Saccorhiza dermatodea* | [EU681421](http://www.sciencedirect.com/science?_ob=RedirectURL&_method=externObjLink&_locator=ncbi-n&_origin=article&_zone=art_page&_plusSign=%2B&_targetURL=http%253A%252F%252Fwww.ncbi.nlm.nih.gov%252Fentrez%252Fquery.fcgi%253Fcmd%253Dsearch%2526db%253Dnucleotide%2526doptcmdl%253Dgenbank%2526term%253DEU681421) | - | - |
| *Sargassum fallax* | GQ368266 | - | - |
| *Sargassum muticum* | [EU681423](http://www.sciencedirect.com/science?_ob=RedirectURL&_method=externObjLink&_locator=ncbi-n&_origin=article&_zone=art_page&_plusSign=%2B&_targetURL=http%253A%252F%252Fwww.ncbi.nlm.nih.gov%252Fentrez%252Fquery.fcgi%253Fcmd%253Dsearch%2526db%253Dnucleotide%2526doptcmdl%253Dgenbank%2526term%253DEU681423) | - | - |
| *Scytosiphon lomentaria* | [EU681424](http://www.sciencedirect.com/science?_ob=RedirectURL&_method=externObjLink&_locator=ncbi-n&_origin=article&_zone=art_page&_plusSign=%2B&_targetURL=http%253A%252F%252Fwww.ncbi.nlm.nih.gov%252Fentrez%252Fquery.fcgi%253Fcmd%253Dsearch%2526db%253Dnucleotide%2526doptcmdl%253Dgenbank%2526term%253DEU681424) | - | - |
| *Scytothamnus australis* | [EU681425](http://www.sciencedirect.com/science?_ob=RedirectURL&_method=externObjLink&_locator=ncbi-n&_origin=article&_zone=art_page&_plusSign=%2B&_targetURL=http%253A%252F%252Fwww.ncbi.nlm.nih.gov%252Fentrez%252Fquery.fcgi%253Fcmd%253Dsearch%2526db%253Dnucleotide%2526doptcmdl%253Dgenbank%2526term%253DEU681425) | - | - |
| *Splachnidium rugosum* | [EU681427](http://www.sciencedirect.com/science?_ob=RedirectURL&_method=externObjLink&_locator=ncbi-n&_origin=article&_zone=art_page&_plusSign=%2B&_targetURL=http%253A%252F%252Fwww.ncbi.nlm.nih.gov%252Fentrez%252Fquery.fcgi%253Fcmd%253Dsearch%2526db%253Dnucleotide%2526doptcmdl%253Dgenbank%2526term%253DEU681427) | - | - |
| *Syringoderma phinneyi* | [EU681429](http://www.sciencedirect.com/science?_ob=RedirectURL&_method=externObjLink&_locator=ncbi-n&_origin=article&_zone=art_page&_plusSign=%2B&_targetURL=http%253A%252F%252Fwww.ncbi.nlm.nih.gov%252Fentrez%252Fquery.fcgi%253Fcmd%253Dsearch%2526db%253Dnucleotide%2526doptcmdl%253Dgenbank%2526term%253DEU681429) | - | - |
| *Tilopteris mertensii* | [EU681430](http://www.sciencedirect.com/science?_ob=RedirectURL&_method=externObjLink&_locator=ncbi-n&_origin=article&_zone=art_page&_plusSign=%2B&_targetURL=http%253A%252F%252Fwww.ncbi.nlm.nih.gov%252Fentrez%252Fquery.fcgi%253Fcmd%253Dsearch%2526db%253Dnucleotide%2526doptcmdl%253Dgenbank%2526term%253DEU681430) | - | - |
| *Undaria pinnatifida* | GQ368267 | - | - |
| *Xiphophora chondrophylla* | GQ368268 | - | - |

^a^Indicates *COI* sequence from [1].

^b^Indicates *COI* sequence from [2].

**References for the Additional file 3**

1. McDevit DC, Saunders GW: **A DNA barcode examination of the Laminariaceae (Phaeophyceae) in Canada reveals novel biogeographical and evolutionary insights.** *Phycologia* 2010, **49**: 235-248.

2. Silberfeld T, Leigh JW, Verbruggen H, Cruaud C, de Reviers B, Rousseau F: **A multi-locus time-calibrated phylogeny of the brown algae (Heterokonta, Ochrophyta, Phaeophyceae): Investigating the evolutionary nature of the "brown algal crown radiation".** *Mol Phylogenet Evol* 2010, **56**:659-674.
