# Supplementary material for: DNA variation in the phenotypically-diverse brown alga Saccharina japonica
Source: BMC Plant Biol. 2012 Jul 11;12:108. doi: 10.1186/1471-2229-12-108 (PMC3490969; doi:10.1186/1471-2229-12-108)
Supplement: Additional file 4 — Coordinates of indels in the rbc LS and ITS gene regions of Saccharina japonica morphological forms. [file 1471-2229-12-108-S4.doc]

**Additional file 4.** Coordinates of indels in the *rbc*LS and *ITS* gene regions of *Saccharina japonica* morphological forms. Coordinates correspond to a 4,286 bp concatenated dataset; see Figure 1.

▲1, a 13-bp deletion of AGTATATCTAAAA (position 1400-1412; *rbc*LS intergenic spacer); ▲2, a 4-bp deletion of CGGT (position 3682-3685; *ITS1*); ▼1, a single nucleotide insertion of T (position 3690; *ITS1*); ▼2, a single nucleotide insertion of T (position 3759; *ITS1*); ▼3, a single nucleotide insertion of A (position 3768; *ITS1*); ▼4, a 4-bp insertion of TTCT (position 4256-4259; *ITS2*); ▲3, a 8-bp deletion of GTAAAAAA (position 4261-4268; *ITS2*).
